# The influence of the gut-brain axis on anxiety and depression: A review of the literature on the use of probiotics

**DOI:** 10.1016/j.jtcme.2024.03.011

**Published:** 2024-03-21

**Authors:** Sara Ferrari, Simone Mulè, Francesca Parini, Rebecca Galla, Sara Ruga, Giorgia Rosso, Arianna Brovero, Claudio Molinari, Francesca Uberti

**Affiliations:** a Laboratory of Physiology, Department of Translational Medicine, University of Piemonte Orientale, Via So-laroli 17, 28100, Novara, Italy; b Noivita srls, spin Off, University of Piemonte Orientale, Via Solaroli 17, 28100, Novara, Italy; c Department for Sustainable Development and Ecological Transition, Italy

**Keywords:** Mood disorders, *Lactobacilli*, *Bifidobacteria*, Postbiotics, Probiotic supplementation

## Abstract

This review aims to argue how using probiotics can improve anxiety and depressive behaviour without adverse effects, also exploring the impact of postbiotics on it. Specifically, probiotics have drawn more attention as effective alternative treatments, considering the rising cost of antidepressant and anti-anxiety drugs and the high risk of side effects. Depression and anxiety disorders are among the most common mental illnesses in the world's population, characterised by low mood, poor general interest, and cognitive or motor dysfunction. Thus, this study analysed published literature on anxiety, depression, and probiotic supplementation from PubMed and Scopus, focusing on the last twenty years. This study focused on the effect of probiotics on mental health as they have drawn more attention because of their extensive clinical applications and positive impact on various diseases. Numerous studies have demonstrated how the gut microbiota might be critical for mood regulation and how probiotics can affect host health by regulating the gut-brain axis. By comparing the different works analysed, it was possible to identify a strategy by which they are selected and employed and, at the same time, to assess how the effect of probiotics can be optimised using postbiotics, an innovation to improve mental well-being in humans.

## Graphical abstract

**Figure:**
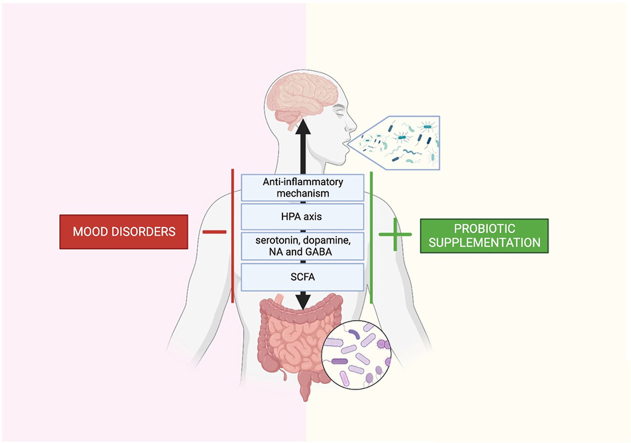

## Abbreviations

5-HT: 5-hydroxytryptamine
ACTH: adrenocorticotropic hormone
BAI: Beck Anxiety Inventory
BDI: Beck Depression Inventory
BDNF: brain-derived neurotrophic factor
CFU: colony-forming units
COVID-19: Coronavirus disease 19
CNS: central nervous system
CRH: corticotropin-releasing hormone
CUMS: chronic unpredictable mild stress
DALY: disability-adjusted life years
GABA: gamma-aminobutyric acid
GAD: generalized anxiety disorder
GLP-1: glucagon-like peptide-1
GSH: glutathione
H2S: hydrogen sulphide
HADS: hospital anxiety and depression scale
HAM-A: Hamilton rating scale for anxiety
HAM-D: Hamilton rating scale for depression
HPA: hypothalamic-pituitary-adrenal
hs-CRP: high-sensitivity C-reactive protein
HSCL-90: Hopkins Symptom Checklist
HSPs: heat shock proteins
IBD: inflammatory bowel disease
IBS: irritable bowel syndrome
IL: interleukin
ISAPP: International Scientific Association for Probiotics and Prebiotics
LPS: lipopolysaccharide
MDD: Major depressive disorder
MGBA: microbiota-gut-brain axis
NA: norepinephrine
NADH: Nicotinamide adenine dinucleotide oxide
NADPH: nicotinamide adenine dinucleotide phosphate oxidase
NO: nitric oxide
NOS: NO synthetase
NPS: neuropsychiatric disorders
OS: oxidative stress
PFC: prefrontal cortex
PLP: pyridoxal 5′-phosphate
PTSD: post-traumatic stress disorder
ROS: reactive oxygen species
SCFA: short-chain fatty acids
SNRIs: serotonin/norepinephrine reuptake inhibitors
SSRIs: selective serotonin reuptake inhibitors
SV2A: glycoprotein 2A in synaptic vesicle
TCAs: tricyclic antidepressants
TGF-β: transforming growth factor-beta
Th1: T helper cells 1
Th2: T helper cells 2
TNF-α: tumour necrosis factor α

## 1 Introduction

Depression is a common chronic condition marked by a depressed state of mind, lack of energy, melancholy, insomnia, and an inability to enjoy life. It can influence thoughts, mood, and physical health.^1^ The term ‘depression’ first entered use in the 19th century, initially referred to as ‘mental depression,’ to describe the lowering of mood, replacing the term melancholy, which, until then, had been used to define mood swings.^2^ According to recent estimates, there are 350 million depressed persons in the world today, and according to the disability-adjusted life years (DALY), depression is the third most common condition and a significant contributor to non-life-threatening disorders.^3^ Furthermore, according to recent research, depression will surpass conditions like cancer and cardiovascular and respiratory disorders to become the most prevalent disease in the world by 2030 due to the ongoing rise in diagnosed instances.^4^ A comprehensive psychiatric interview conducted by a medical professional is the best baseline test for detecting depressive symptoms. Still, it is too time-consuming and, therefore, uneconomical for many patients with major depressive disorder (MDD) who go undiagnosed and do not receive adequate treatment.^5^ For this reason, numerous rating scales have been developed over the years.^6^^,^^7^ Based on the results obtained, the choice of treatment for the patient depends on the presence of severe depression, which may require more drastic treatment choices.

Anxiety is a mental condition brought on by a threat or perceived threat; although it is a standard component of life, excessive or improper anxiety can lead to sickness.^8^ Among the most common psychiatric disorders, anxiety disorders such as generalized anxiety disorder (GAD), panic disorder/agoraphobia, social anxiety disorder, and others are frequently underreported and undertreated. Treatment is only necessary when a patient exhibits considerable suffering or has problems related to the disease.^9^ Since the beginning of modern psychiatry in the late 18th century, numerous authors have described the phenomenology of GAD, which is persistent anxiety accompanied by apprehension or worry about many aspects of daily life. Between the 19th and 20th centuries, “pantophobia” and “anxiety neurosis” were used to diagnose generalized anxiety. This terminology described the paroxysmal symptoms (panic attacks) and the anxious frame of mind.^10^ One of the most prevalent kinds of illnesses in the world and the sixth major cause of disability is anxiety disorders.^11^ For example, an estimated 40 million adults in the United States have an anxiety disorder, with a 41.7% risk of developing a related condition in their lifetime. These chronic disorders significantly impact the quality of life, making it challenging to adapt to society. In addition, anxiety disorders are associated with psychiatric and physical comorbidities, increased utilization of medical services, and significant social costs associated with loss of productivity and work impairment.^12^

The difficulty in finding causes involved in the onset of depression has led to more significant efforts to understand the brain circuits underlying this disorder. Much of this research has focused on the prefrontal cortex (PFC) and the amygdala, as they are primarily interconnected and work to synchronise the expression of emotions.^13^ The PFC inhibits the amygdala's activity under physiological circumstances, reducing its output and preventing the manifestation of improper emotions. Prefrontal control, however, degenerates under adverse events, such as prolonged exposure to unavoidable stress that may trigger the onset of psychiatric conditions (such as anxiety disorders and depression), leading to aberrant amygdala activation and deficits in emotion and behaviour.^17^ In more detail, during anxiety states, these regions show structural and functional changes potentially triggered by altered glutamatergic and gamma-aminobutyric acid (GABA) transmission.^14^

Additionally, a recent positron emission tomography study found that depressive patients had lower amounts of glycoprotein 2A in synaptic vesicles (SV2A), indicative of fewer synapses.^15^ In post-mortem investigations of depressed people, analysis of the decline in synaptic markers and the number of synapses in the PFC has revealed direct evidence of synaptic activity loss.^16^ Thus, despite the abundance of research on this disorder and the various hypotheses that have been advanced, the aetiology of depressive disorder is still not fully understood.^17^

### 1.1 The common risk factors for mood disorders

Several risk factors may be associated with the development of disorders such as depression or anxiety disorders; specifically, MDD is one of the most prevalent and disabling mental disorders. The probability of ongoing depressive episodes is increased by 69% when a person has a history of MDD, which must be considered.^18^ Moreover, individuals with a family history of MDD are three to five times more likely to develop MDD.^19^

Regarding depression, the following risk factors have been identified in the last 40 years of research: nervous system dysfunction such as disturbances in sleep, concentration/attention, suicidal thoughts,^20^ feelings of guilt or thoughts of worthlessness,^21^ depressed mood; stress factors like changes in energy/fatigue; certain sociodemographic factors like female subjects; parental depression and behavioural patterns including changes in appetite and weight.^22^

It is essential to consider that, although cognitive factors are fundamental, how one subjectively perceives the surrounding environment is among the triggers for depression, as some individuals are inclined to attach more importance to specific negative experiences as if they were more significant than other events.^22^ Furthermore, it should be emphasised that subjective but distorted perceptions of stress can theoretically trigger depression even following a minor or imagined event.^23^ Individuals may have distinct risk factors for specific anxiety disorders; for instance, GAD and phobias are more prevalent in women, although social anxiety affects both men and women equally.^24^ However, there are some common risk factors for all anxiety disorders, such as personality traits, traumatic events that happened when one was a young child or an adult, and a family history of anxiety or other mental disorders. Furthermore, anxiety disorders and MDD often occur concurrently and share a wide range of risk factors.^25^

### 1.2 Negative consequences on quality of life

It is important to emphasise that MDDs are widespread in the global population, contributing substantially to the worldwide disease burden and leading to a substantial increase in healthcare expenditure.^26^^,^^27^ Depression is also correlated with increased mortality as it causes high suicide rates: it is responsible for approximately 40,000 cases of suicide each year in the United States, and the highest suicide rate occurs in older men.^28^ In addition, even if effective pharmacological treatment is available, almost 50% of sufferers may initially fail to respond to treatment; moreover, complete remission is uncommon while relapses are recurrent.^29^ It should also be considered that anxiety disorders cause great suffering to patients and those around them and are costly in terms of increased medical care utilization. Additionally, anxiety disorders have a very high morbidity rate, which includes drug and alcohol addiction, which raises the risk of unfavourable cardiac events.^20^

### 1.3 Prevention and therapy of mood disorders

Depression has long been acknowledged by psychology and psychiatry as a key management issue. Therefore, developing long-lasting preventive therapies that can impact public health is one of the sector's key goals,^30^ since up to 20% of young people have a depressive episode or an anxiety disorder by turning 18.^31^ In this context, the school environment frequently provides a wide range of universal preventative actions for youth.^32^ On the other hand, tailored prevention measures are aimed at persons with a more significant risk profile for the disorder, such as familial risk or poverty (chosen prevention) or those who demonstrate subclinical indications (indicated prevention).^33^ Preventive actions have a wide range of advantages. The first benefit is that therapy can occasionally prevent an illness from arising; for example, research shows that 22% of new occurrences of depression each year can be prevented.^34^ Secondly, initiating preventative programmes from a young age, when behaviour is most adaptable, will probably yield more significant outcomes than treating patients with rigid patterns of cognition and behaviour that are already established and entrenched.^35^ While it is encouraging that targeted and appropriate preventive therapies can effectively manage anxiety and depression and are valuable additions to the existing mental health support system, more research concerning this is required.^36^ Furthermore, innovation in psychopharmacology is essential because average short-term benefits are typically modest, and the long-term balance of benefits and hazards is generally underestimated.^37^^,^^38^ The commonly prescribed pharmacological interventions for the treatment of depression encompass tricyclic antidepressants (TCAs), selective serotonin reuptake inhibitors (SSRIs) such as citalopram and escitalopram, serotonin/norepinephrine reuptake inhibitors (SNRIs) including venlafaxine, desvenlafaxine, and duloxetine, as well as atypical antidepressants like bupropion, mirtazapine, and nefazodone.^20^ As a result, classical therapy improves serotonin or norepinephrine (NA) availability at postsynaptic levels.^17^ Indeed, the first-line therapy includes SSRIs (fluoxetine, sertraline, paroxetine, escitalopram, and citalopram) or TCAs (amitriptyline, imipramine, and nortriptyline) with serious side effects.^39^ Furthermore, continued usage may require greater doses to have the same effect, leading to tolerance and dependence problems.^20^

Although there are several pharmacological treatments for MDD, due to a lack of funding, antidepressants are utilized more frequently than psychosocial approaches.^37^ Indeed, psychotherapies that successfully treat anxiety and depression include cognitive-behavioural and interpersonal therapy, which can be used with medication. These types of treatment emphasis behavioural skill development to help patients act and react to anxious and depressing situations more flexibly.^40^ For example, the side effect of weight gain, which can cause several comorbidities and shorten life expectancy, is also a significant public health concern associated with antidepressants that may contribute to metabolic risk by increasing hunger and reducing feelings of fullness.^41^

### 1.4 Natural extracts in mood disorders

Due to the widespread search for effective natural anxiolytic and antidepressant treatments, such as herbal remedies and dietary supplements, with a lower risk of side effects, complementary and alternative medicines have been used more frequently in recent years.^42^ These treatments include dietary supplements and herbal remedies. An example is the herbaceous perennial plant *Hypericum perforatum*, used in Chinese traditional medicine and in many European countries to treat depression. According to numerous studies, it has clinical efficacy for reducing depressive symptoms and has effects comparable to standard SSRIs.^43^ Furthermore, polyphenols have been shown to reduce the expression and activity of transcription factors, protein complexes, and pro-inflammatory cytokines that lead to neuroinflammatory reactions. These exercises might lessen the behavioural symptoms of depression.^44^^,^^45^ For instance, numerous meta-analyses have validated the antidepressant properties of curcumin, which have been demonstrated in both animal and human trials.^46^^,^^47^ The antidepressant properties of silymarin, a polyphenol that boosts neurotransmitter levels, promotes neurogenesis, and prevents the activation of the oxidative stress,^48^ have also been examined. Quercetin,^49^ was associated with an increase of BDNF mRNA in some brain structures It is appropriate to include psychobiotics, which are living bacteria that colonise the intestinal flora. On brain functions, they have both immediate and indirect positive effects. These bacteria exhibit anxiolytic and antidepressant activities, although at low doses, according to a preliminary study.^50^ According to recent literature,^51^^,^^52^ psychobiotics are the cornerstone of the theory of a physical and biochemical link between the gut and brain that can alter neurotransmitters and proteins like GABA, serotonin, glutamate, and brain-derived neurotrophic factor (BDNF), which are essential for regulating excitatory–inhibitory neural balance, mood, cognitive abilities, and learning and memory processes.

To thoroughly explain the utilization of probiotic supplementation, the gut-brain axis, and the biochemical pathways triggered to modify mental diseases, both topics are covered in detail in the following paragraphs.

## 2 Information gathering

This research aimed to identify guidelines for the probiotics used and identify the options and protocols of administration to explore a possible new approach. The topic of this narrative review includes studies present in the literature about the effects of probiotic supplementation to ameliorate anxiety and depression symptoms.

In this regard, papers from different databases such as PubMed and Scopus were selected using the following keywords: anxiety and probiotic supplementation; depression and probiotic supplementation; mood disorders and probiotics supplementation. The research from this preliminary screening revealed over 300 papers subdivided for each term, such as anxiety and probiotic supplementation (n = 147 articles), depression and probiotic supplementation (n = 184 articles), mood disorders and probiotics supplementation (n = 44 articles). The number of studies (humans without distinguished age or sex of the populations, *in vitro* and *in vivo*) about the topic was reduced by applying other inclusion criteria: (a) they had to address probiotic supplementation, depression, and anxiety; (b) they had to be written in English; (c) they had to assess how interventions affected anxiety and depressive symptoms or biomarkers; (d) they had to evaluate anxiety or depression in clinical studies through these scales: Beck Anxiety Inventory (BAI), Beck Depression Inventory (BDI) and hospital anxiety and depression scale (HADS); (e) the timeline of the published article considered was from 2002 to 2024; (f) the journal of each article selected must be included in Q1 racking for at least one of these thematic area: Medicine (miscellaneous), Psychiatric and Mental Health, Biological Psychiatric, Clinical Phycology, Neurosciences (micellaneous), Biochemistry Genetics and Molecular Biology (micellaneous), Nutrition and Dietetics, Food sciences, Immunology, Microbiology, Gastroenterology.

In addition, once duplicates had been eliminated, the titles and Abstracts were explored to exclude the studies that weren't compatible with the review's criteria. This article screening was used to prepare all manuscript Sections.

In addition, the present narrative review also included four links within the inclusion criteria and five books presented outside published with the older findings before 2000 relative to the discovery of probiotics.

**Fig. 1:**
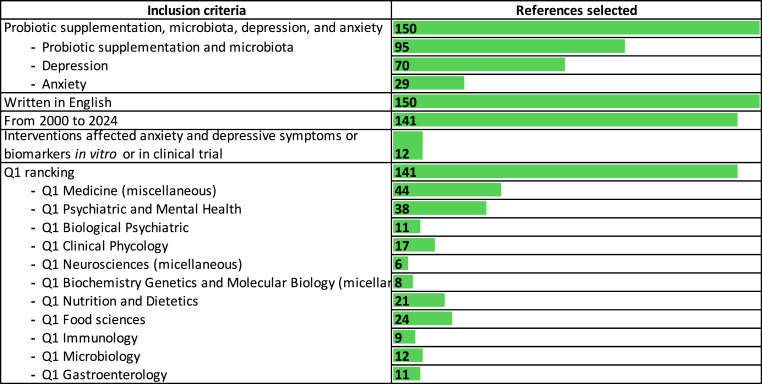
Inclusion criteria of the studies analysed and their division into categories.

Based on these criteria, the selection includes the following (Fig. 1):

## 3 The role of the gut-brain axis in mood regulation

**Fig. 2:**
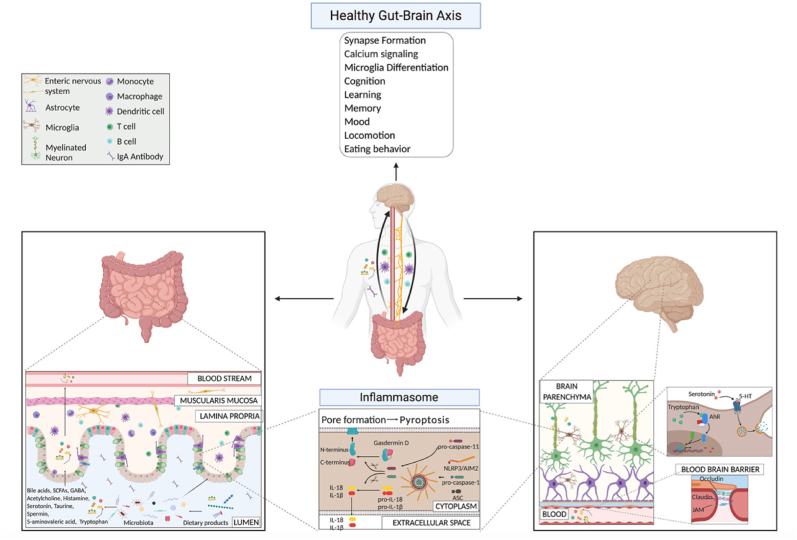
Gut-brain axis mechanisms under physiological conditions highlight microbial products.^53^

The vagus nerve, the immune system, metabolites, and bacterial products are parts of the gut-brain axis, a two-way network of signalling channels faciliting communication between the neurological and gastrointestinal tract (Fig. 2).^53^ The gastrointestinal tract contains a variety of bacteria and their metabolites, which can have a major impact on various biological processes in the organism.^54^ Furthermore, the brain regulates intestinal transit, motility, secretion, and permeability via the autonomic nervous system, which affects the composition and functionality of the gut microbial population.^54^

The brain-gut-microbiota axis is regulated by several mechanisms, including the hypothalamic-pituitary-adrenal (HPA) axis.^55^ The hypothalamus releases corticotropin-releasing hormone (CRH) in a humoral manner in response to stress.^55^ CRH then travels through the bloodstream to the pituitary gland, where it stimulates the creation of adrenocorticotropic hormone (ACTH), which in turn causes the adrenal glands to release glucocorticoid steroid hormones (stress hormones) like cortisol or corticosterone.^56^ Stress hormones promote the disintegration of tight junctions, thereby enhancing intestinal barrier permeability at the systemic level.^57^ The HPA axis and immune system are subsequently activated due to bacterial translocation.^55^ As a result, it is possible to pinpoint the human microbiota's critical function in controlling the body's homeostasis.

### 3.1 Dysbiosis of human intestinal microbiota

Dysbiosis is defined as an imbalance in the composition and operation of the resident microbial community of the enteric tract several studies have associated with several diseases of gastrointestinal tract like inflammatory bowel disease (IBD),^58^ irritable bowel syndrome (IBS).^59^ Although it is still unclear whether intestinal microbial dysbiosis directly causes inflammation in IBD or merely contributes to it by establishing an unfavourable environment in the gastrointestinal tract, there is growing evidence that it plays a role in the pathophysiology of the illness.^58^ Although the changes are inconsistent, differences in the microbiota's composition across the different illness subtypes and healthy individuals have been identified about IBS.^59^ The microbiota composition has also been associated with celiac disease, with richer and more varied changes than control individuals.^60^

The structure of the host's intestinal epithelium, peristalsis, dietary changes, age, genes, temperature, interactions between various bacterial species, immune system responses—especially those of T and B cells—use of antibiotics or radio- and chemotherapeutic drugs, environmental and physical stress, and finally psychological stress are some of the factors that affect the microbiota's composition.^61^ In this situation, dysbiosis alters the intracellular tight junctions that protect the intestinal mucosa's integrity and permeability, which is required to thwart pathogen infiltration at the systemic level.^62^ In addition, the gut microbiota has a regulatory role in oxidative stress (OS). When nitric oxide (NO) and nitrite concentrations have risen due to high nitrate intake, *Lactobacilli* and intestinal *bifidobacteria* can transform nitrate and nitrite into NO while enhancing its release by host epithelial cells. Through their NO synthetase (NOS), intestinal Bacilli and *Streptomycetes* also create NO in addition to nitrates.^63^ Even though NO has a role in signalling and apoptosis at nanomolar concentrations, excessive NO synthesis is dangerous since it is connected to neuroinflammation, cellular harm, and neurodegenerative disorders.^63^ The gut microbiome is, in turn, affected by a diverse range of metabolites, including gaseous mediators such as hydrogen sulfide (H_2_S) that bacteria use as an energy source in the gut.^64^

## 4 Dysbiosis and brain homeostasis

**Fig. 3:**
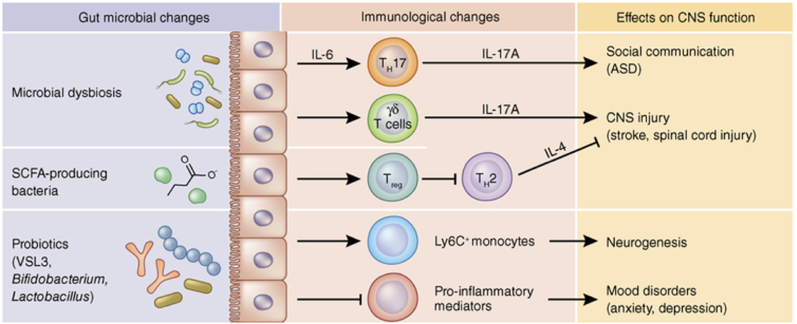
Effects of the microbiota on peripheral immune cells and CNS function. Image readapted from Fung et al. study^73^ **ASD**: autism spectrum disorder; **CNS**: central nervous system; IL**-4**: interleukin-4; **IL-6**: interleukin-6; **IL-17A**: interleukin-17A; **SCFA**: short-chain fatty acid.

Dysbiosis has the potential to induce an inflammatory state that may disseminate into the bloodstream and subsequently infiltrate the brain 65. Undoubtedly, the significance of inflammation should not be undervalued, as specific data substantiates its pivotal involvement in various chronic illnesses, including depression.^66^ Besides cytokines, other mediators can transmit signals from the gastrointestinal tract to the brain through the vagus nerve. Following the consumption of the enteroendocrine cells were stimulated to produce glucagon-like peptide-1 (GLP-1).^67^ Various modified conditions can contribute to developing gut dysbiosis-induced neuropsychiatric symptoms. These conditions involve changes in microbial composition and metabolite production, which subsequently impact the levels and synthesis of neurotransmitters like serotonin, dopamine, NA, and glutamate. Consequently, dysbiosis causes an imbalance of the gut-brain axis with negative consequences on brain mechanisms.^68^ Evidence supports the notion that microorganisms can generate neuroactive chemicals, which directly facilitate communication between the gastrointestinal tract and the central nervous system (CNS). For example, the bacteria belonging to the *Lactobacillus*, *Bifidobacteria*, *Enterococcus*, and *Streptococcus* species can impact the physiological functioning of brain cells through the production of neurotransmitters such as acetylcholine, GABA, and serotonin 69,70. In the present context, it has been demonstrated that *Lactobacillus rhamnosus* can regulate the expression of the GABA receptor within the brain, hence exhibiting a favourable impact on managing mood disorders.^71^ The disorder known as permeable bowel syndrome, which is caused by increased intestinal permeability due to altered dysbiosis, is also characterised by decreased expression of tight junction proteins which increases secretion of pro-inflammatory molecules.^72^ Moreover, an imbalance in the functioning of the gastrointestinal system leads to the initiation of persistent systemic inflammation, which disrupts the structural integrity of the blood-brain barrier by causing degradation of tight junction and anchor proteins in specific regions of the frontal cortex, hippocampus and striatum. As a result, this disruption in brain function occurs.^73^^,^^74^ The elevation in permeability of the blood-brain barrier leads to an excessive movement of immune cells and harmful microbial metabolites into the brain. This, in turn, amplifies the release of cytokines, chemokines, and endocrine messengers associated with stress within the brain parenchyma.^75^ Indeed, the imbalance in gut bacteria disrupts the signalling between the stomach and the brain, activating the pro-inflammatory process (Fig. 3). This activation is directly associated with symptoms related to neuropsychiatric disorders NPS.^76^

### 4.1 Gut microbiota and depression

Reduced expression of tight junction is another sign of a changed dysbiosis. Multiple studies have documented a disparity in the levels of pro-inflammatory cytokines, including interleukin (IL) −1, IL-6, IL-8, IL-12, and tumour necrosis factor α (TNF-α), and the levels of anti-inflammatory cytokines, such as transforming growth factor-beta (TGF-β) and IL-10, in individuals diagnosed with depression.^77^ As previously stated, the biochemical activation of the inflammatory pathway is distinguished by an excessive generation of reactive oxygen species (ROS). The existence of OS is substantiated by identifying elevated levels of lipid peroxidation by-products, such as malondialdehyde and 4-hydroxynonenal, in individuals diagnosed with depression.^78^ A changed microbiota has the potential to activate nicotinamide adenine dinucleotide phosphate oxidase (NADPH) and NO production, hence initiating OS. In typical circumstances, the intestinal epithelial barrier is safeguarded against OS and inflammation by a group of intracellular proteins known as heat shock proteins (HSPs), which are widely distributed.^79^ On the other hand, the liberation of these substances into the extracellular matrix, a process that occurs during cellular necrosis or apoptosis, can have notable deleterious effects. Extracellular HSPs have been shown to elicit an inflammatory response, resulting in a heightened release of pro-inflammatory cytokines.^80^ In addition, individuals with chronic depression exhibit increased levels of IgA immunoglobulins and IgM antibodies against lipopolysaccharide (LPS) derived from *Enterobacteriaceae* in their plasma. This suggests that the microbiota may contribute to depression by potentially promoting a persistent state of inflammation.^81^

### 4.2 Gut microbiota and anxiety disorder

There is a clear correlation between inflammation of the gastrointestinal system and the expression of anxiety. Several neuropeptides controlled by the microbiome, such as dopamine, NA, GABA and serotonin, among others, can influence brain function and modulate the composition and function of the gut microbiota, reflecting the complex bidirectional crosstalk between the gut and brain^82^ Furthermore, a study revealed a significant decrease in the richness and diversity of the microbial population in individuals diagnosed with GAD. This decrease was accompanied by a lower prevalence of bacteria that produce short-chain fatty acids (SCFA) and an increased prevalence of *Escherichia-Shigella*, *Fusobacterium*, and *Ruminococcus gnavu*.^83^ SCFA are typically present in the intestinal tract; nonetheless, they can enter the systemic circulation and traverse the blood-brain barrier via transporters situated on the vascular epithelial cells of the brain. Neuroglial cells play a significant role within the brain since they can influence many neurotransmitters, including glutamate, glutamine, GABA, and neurotrophic factors.^84^

Moreover, gut dysbiosis resulting from bacterial infection can intensify anxiety by affecting the immune and metabolic pathways within the gut-brain axis. For instance, previous research has established that infection with *Campylobacter jejuni* leads to heightened depressive or anxious behaviour by triggering the activation of c-Fos proteins, which serve as indicators of neuronal activation.^85^, ^86^ Eventually, the available research has indicated a strong and reciprocal relationship between disruptions in the gut microbiome and psychiatric illnesses. Numerous investigations conducted on animal models and human subjects have shown a potential advantageous impact of probiotics, including *Lactobacillus* and *Bifidobacterium*, on anxiety and depression.^86^

## 5 Different role of probiotics: a general overview

The term “probiotic”, derived from the Latin word “pro” and the Greek word "βιoσ," meaning “for life,” was coined by Werner Kollath, a German scientist, in 1953. Kollath used this term to refer to the “active substances” crucial for promoting living organisms' proper growth and well-being. In 1965, Lilly and Still well employed this terminology in an alternative context to denote “substances emitted by an organism that induce the development of another organism.” In 1992, Fuller defined probiotics as “a dietary supplement containing live microorganisms that exert a beneficial effect on the host by enhancing the microbial equilibrium within the gastrointestinal tract”.^87^ In detail, *Lactobacillus*, *Bifidobacterium*, and *Saccharomyces boulardii* are the probiotics most frequently employed in various applications. *Lactobacillus* and *Bifidobacterium* are classified as Gram-positive facultative anaerobic obligate bacteria, but *S. boulardii* is categorised as a yeast organism. The *Lactobacillus* genus comprises multiple species, such as *L. acidophilus*, *L. rhamnosus*, *L. bulgaricus*, *L. reuteri*, and *L. casei*. At the same time, the *Bifidobacterium* species frequently employed in probiotic formulations encompass *B. animalis*, *B. infantis*, *B. lactis*, and *B. longum*. It is essential to highlight that not all probiotic species are inherent to the typical human gut flora. Thus, the positive effects associated with one strain cannot be extrapolated to other strains.^88^ The historical utilization of probiotics precedes the understanding of microorganisms. For instance, ancient Egyptian hieroglyphics portrayed the consumption of fermented milk products, while Tibetan nomads historically employed fermented yak milk to preserve milk throughout extensive travels.^89^ During the 19th century, researchers observed the perceived impact on human health using fermented dairy products. Nevertheless, the underlying cause of these health consequences remained unidentified. In 1905, Elie Metchnikoff, a former collaborator of Pasteur in the 1860s, attributed the extended lifespan observed in Bulgarian rural populations to the presence of *Lactobacilli* used in the fermentation process of yoghurt, rather than the yoghurt itself. These *Lactobacilli* are typically found in the colon and play a role in mitigating the detrimental impacts of gastrointestinal metabolism, which can contribute to disease and the ageing process.^90^ After these findings, researchers investigated the effects of *Lactobacillus* on human subjects. One of the initial inquiries in this domain was conducted in 1922, wherein *L. acidophilus* was administered to a cohort of 30 individuals afflicted with chronic constipation, diarrhea, or eczema. The results demonstrated amelioration in all three conditions. In addition, a study conducted in 1932 confirmed the impact of *L. acidophilus* on individuals suffering from constipation and mental illness.^91^ During the period spanning from the 1950s to the 1980s, the field of probiotic research mainly concentrated on examining various strains that might be obtained from natural sources or derived from human hosts, with a particular emphasis on identifying their methods of action. Further investigation has contributed to the development of knowledge regarding the intricate dynamics between the indigenous microbial communities and their capacity to withstand the infiltration of harmful bacteria, commonly known as colonisation resistance.^87^ An observable trend since the 2000s has been the exponential growth of clinical research examining the effectiveness and safety of these products.^92^ The Human Microbiome Project conducted metagenomic research, employing DNA sequencing techniques to examine bacterial populations. This investigation successfully discovered over 40,000 distinct species residing in the colon and generated comprehensive profiles of the typical microbiological components seen in individuals who are in good health.^93^ Furthermore, a comprehensive probiotic data analysis was conducted in 2014. The study established three overarching classifications: the first category encompassed dietary supplements without any health claims, which are generally considered safe and do not require evidence of efficacy. The second category comprised dietary supplements with specific health claims, supported by evidence-based efficacy derived from clinical studies or meta-analyses and intended for enhancing natural defences or alleviating symptoms. Lastly, the third category consisted of probiotic drugs, which necessitated clinical studies demonstrating efficacy for specific indications or diseases, along with a justification of the risk-benefit ratio and adherence to regulatory standards for drugs.^94^

As previously stated, the probiotic bacteria that are most frequently utilized are *Lactobacillus* and *Bifidobacterium*. When oral probiotics are consumed, they traverse the gastric tract and attach to the intestinal mucosa, competing with pathogenic bacteria in epithelial adhesion. Probiotics have been found to exhibit antimicrobial properties through the production of compounds that can combat pathogens. Additionally, they can reduce pH levels by generating lactic acid, creating an unfavourable environment for pathogenic growth. Probiotics also compete with pathogens for adhesion, colonisation, nutrients, and other essential factors within the gut. Furthermore, they can impede the growth of pathogens by directly binding to Gram-negative bacteria.^66^ The dynamic nature of gut microbial communities allows for their modification through various influences, such as lifestyle choices, dietary patterns, and the administration of antibiotics. Consequently, probiotics have emerged as a viable intervention for managing and preventing diverse gastrointestinal disorders, including IBD.^95^ For example, various multi-omics investigations have examined the involvement of *Bacteroides, Faecalibacterium prausnitzii, Ruminococcus* spp., and *Bifidobacteria* in IBS and their response to treatment. These studies have identified distinct changes in metabolite profiles and have revealed increased immune and inflammatory pathways in IBS patients.^96^ Nevertheless, a notable degree of heterogeneity was seen throughout the studies, and no consistent gut microbiota features associated with IBS were identified.

## 6 Multi-strain probiotics effectiveness

When preventing or treating depression, even as supplemental therapies, probiotics in combination may be more helpful than single bacterial strains; the strains' type, combination, and origin (human versus non-human) all have a role in determining efficacy at the best level.^97^

Recent studies have also discovered that probiotic mixtures produce better health results than single strains. They mixed probiotic strains of *Lactobacillus* and *Bifidobacterium* spp*,* due to their significant anti-depressive capabilities.^98^ An eight-strain probiotic formulation containing bacteria such as *Bifidobacterium*, *Lactobacillus*, and *Lactococcus* has been shown to reduce symptoms of depression, anxiety, and stress in humans.^99^^,^^100^ Indeed, probiotic supplementation with *L. helveticus* R0052 and *B. longum* R075 in combination over 30 days significantly improved mood and overall psychological well-being in 66 healthy human volunteers compared to the single microorganism, as measured by a few self-reported measures, including HADS. It reduced the amount of free cortisol in the urine, possibly attenuating the HPA axis.^101^ The treatment of young adults between the ages of 18 and 24 who were under test stress with a 28-day course of a multi-probiotic strain composed of *B. coagulans* Unique IS2, *L. rhamnosus* UBLR58, *B. lactis* UBLa70, *L. plantarum* UBLP40, *B. breve* UBBr01, and *B. infantis* UBBI01 produced exciting results in terms of the combination of several probiotics. This treatment not only improved the result of effort but also significantly reduced serum cortisol levels.^97^

Similar to this, patients with mild to moderate depression who received a multi-species probiotic mix for 4 weeks, as opposed to a single strain, containing *B. breve* CCFM1025, *B. longum* CCFM687, and *Pediococcus acidulating* CCFM6432, have reported a significant improvement in depressive symptoms as well as in gastrointestinal functions. Unexpectedly, these effects did not appear to be related to modifications in gut microbiota composition, suggesting a different mode of action. Psychological symptoms significantly improved following 4 or 8 weeks of multi-species probiotic administration, according to the results of separate randomized, double-blind, placebo-controlled trials.^102^ Furthermore, the PROVIT study, a randomized controlled trial conducted in Austria, discovered that consuming a multi-strain probiotic for 4 weeks increased the relative abundance of *Ruminococcus gauvreauii* and *Coprococcus*. These strains are recognised for their ability to produce butyrate, an SCFA that possesses immunoregulatory and anti-inflammatory properties. Butyrate plays a crucial role in maintaining the integrity of the intestinal epithelial barrier and preventing inflammation in the brain 103.

## 7 Use of probiotics to modulate anxiety and depression

**Fig. 4:**
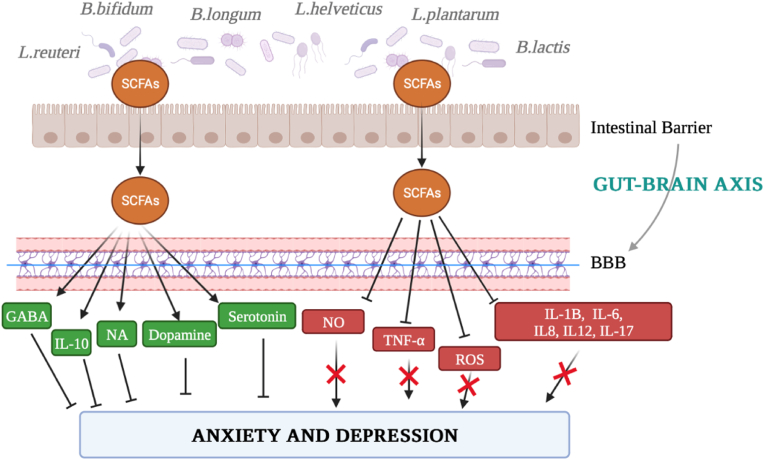
Schematic picture of the gut-brain axis and the mechanisms of action of probiotic supplementation. **BBB**: blood-brain barrier; **GABA**: gamma-Aminobutyric acid; **IL-1B**: interleukin-1B; **IL-6**: interleukin-6; **IL-8**: interleukin-8; **IL-10**: interleukin-10; **IL-12**: interleukin-12; **IL-17**: interleukin-17; **NA**: norepinephrine; **NO**: nitric oxide; **ROS**: reactive oxygen species; **SCFA**: short-chain fatty acid; **TNF- α**: tumour necrosis factor α.

Probiotic use has skyrocketed recently, and pathological diseases have been alleviated by probiotic-mediated microbiota regulation towards the microbiota-gut-brain axis (MGBA). Although research is still in its early phases, clinical and preclinical studies have shown the potential of probiotics in the context of mental health by creating the groundwork for applying preclinical models to people. Probiotic intervention may help treat potential aetiopathological causes of depression and anxiety that also involve inflammation, neurotransmitters, the HPA axis, and epigenetic mechanisms because probiotics have anti-inflammatory properties and help to restore levels of neurotransmitters involved in the onset of depression-like serotonin and dopamine, NA, and GABA (Fig. 4).^104^, ^105^, ^106^ Since animal models are primarily employed to assess the safety of probiotic bacteria and potential mechanisms of action, the initial step in understanding probiotic strains was carried out through *in vivo* investigations to prove their health benefits.^107^

### 7.1 Preclinical *in vivo* studies

In a preliminary study by Messaoudi et al.,^101^ the psychotropic effects of a probiotic preparation containing *L. helveticus* R0052 and *B. longum* R0175 were examined in rats. Specifically, the probiotic preparation contained a combination of *L. helveticus* R0052, and *B. longum* R0175 dissolved in a 0.9% NaCl solution. On the fourteenth day, a probe was put inside the cages, and when the mice touched it, it shocked them with a mild electric current. To support the compound's anti-anxiety properties, the global stress/anxiety score results were determined.^101^ The research team used the same formulation in a double-blind, controlled, randomized, 30-day study including human participants. Participants had blood collection as part of the pre-test medical check-up to ensure their biological safety parameters were within normal ranges. Due to reduced levels of somatization, sadness, and anger-hostility in the probiotic formulation group's patients compared to the control group's patients over time, the Hopkins Symptom Checklist (HSCL-90) overall severity index was lower in the probiotic formulation group's patients. Additionally, the probiotic-treated patients had a lower self-blame score. They were reported to be more problem-solving-focused than controls, indicating a difference between the two groups in emotional reactivity. These investigations showed that consumption of *B. longum* R0175 and *L. helveticus* R0052 reduced psychological distress without causing adverse side effects.^101^ However, these data are preliminary, so further preclinical and clinical investigations should be extended to examine more specific aspects.

Similarly, the research conducted by Ramalho et al. provides evidence for the depressive and anxiolytic properties of probiotics. This was achieved through a series of tests to assess the potential effects of *L. lactis subspecies cremoris* LL95 in mice.^108^ To simulate the vulnerability of females to the onset of mood disorders, the study was conducted *in vivo* on 90-day-old female mice. The findings revealed that *L. lactis* LL95 treatment had no adverse effects on any of the mice's locomotor activity during the study or the Open-Field Test, nor did it cause any mice to exhibit increases in depression levels. *L*. *lactis* LL95 has also demonstrated a free radical scavenging effect, suggesting it may be a possible therapy. Treatment with L. *lactis* LL95 also decreased depressive behaviour in mice. The observations from this mouse model also indicate that oral supplementation with *L. lactis* LL95 can potentially reduce symptoms of anxiety and depression.^108^ Further research is necessary to fully understand the molecular mechanisms behind the benefits observed since it is impossible to rule out the participation of additional processes and functional systems that may also be involved in *L. lactis* LL95's beneficial effects.

In a different study, the probiotic *Faecalibacterium prausnitzii*'s anxiolytic and antidepressant properties were investigated to gauge how much chronic unpredictable mild stress (CUMS) in rats was reduced. The investigation was divided into two phases: the CUMS procedure period, which lasted from weeks 3–6, and the recovery period, which lasted from weeks 8–11. Several low-intensity stressors were applied to the rats, and the stress technique was unpredictable because it changed daily. Throughout the 4-week trial, these stressors were repeated for 2 h each day for 1 week on a randomly assigned schedule. According to the findings, adding *F. prausnitzii* to a diet had preventive and therapeutic effects on depression and anxiety brought on by CUMS. It prevented the effects of CUMS on the release of corticosterone, C-reactive protein, and IL-6 by increasing levels of SCFA in the cecum and cytokines such as IL-10 in plasma. According to the findings of this investigation, *F. prausnitzii* exhibits considerable promise as a probiotic agent.^109^

All these findings are summarized in Table 1.

**Table 1:** Summary of selected *in vivo* studies.

Study dimension and population	Treatment	Methodology	Results	Ref
36 Male Wistar HsdBrlHan rats	Daily administration of a probiotic combination of *L. helveticus* R0052 and *B. longum* R0175 for 2 weeks	• The animals were exposed to a 2 mA electric shock when the forepaws touched the probe, and its behaviour was recorded for 5 min.	• Significantly reduction of anxiety-like behaviour	^107^
12 Female C57BL/6 mice	Daily administration of 10^9^ CFU of *Lactococcus lactis* subsp. *cremoris* LL95 for 28 days	The animals were subjected to: • Open-Field Test; • Elevated Plus Maze Test; • Tail Suspension Test; • Forced Swim Test; The hippocampus of mice was removed and utilized for: • ROS quantification; • Ferric reducing antioxidant power assay.	• Improvement of depressive- and anxiety-like behaviour; • Decrease of immobility time in the tail suspension test and forced swim test; • Increase of time spent in the open arms on the elevated plus maze.	^108^
60 Male Sprague-Dawley rats	Daily administration of 1 × 10^9^ CFU of *F. prausnitzii* for 4 weeks	The animals were subjected to: • Open-Field Test; • Elevated Plus Maze Test; • Forced Swim Test; The cecum and the blood of mice were utilized for: • SCFAs concentration in the cecum; • Corticosterone, C-reaction protein (CRP) and cytokine determination in the blood.	• Higher levels of SCFAs; • Higher levels of IL-10 in the plasma that prevented the effects on corticosterone, C-reaction protein and IL-6 release.	^109^

### 7.2 Clinical investigation

To date, several studies concern the effectiveness of single probiotic strains in modulating mood disorders in humans. However, of these studies, only those using BAI, BDI or HADS were considered, as they have been a mainstay in mental health assessment for many decades. For instance, in a randomized, double-blind, placebo-controlled research involving 44 patients with IBS and diarrhea and mild to moderate anxiety and depression, assessed with HADS evaluation, the effects of *B. longum* NCC3001 on anxiety and depression in individuals with IBS were evaluated.^110^ This study demonstrated that supplementing with *B. longum* NCC3001 for 6 weeks had no appreciable impact on symptoms of anxiety or IBS; however, patients receiving this treatment had higher quality-of-life scores and less sensitivity to distressing emotional stimuli in several brain regions, including the amygdala and frontal-limbic areas, compared to those receiving a placebo. Even if the probiotic group's urine methylamine and aromatic amino acid metabolite levels were lower than those of the placebo group, the probiotic group's fecal microbiota profiles, serum inflammatory markers, and levels of neurotrophins and neurotransmitters were comparable. This study, therefore, demonstrated how *B. longum* NCC3001 could lower depression scores in IBS patients while not affecting anxiety levels and improving quality of life.^110^ However, no effect of supplementation on individual bowel symptoms was demonstrated, as the study was inadequate to detect these changes. Therefore, a larger study in patients with IBS and depression in comorbidity is needed to verify the data on psychiatric and intestinal symptoms before this supplementation can be recommended in clinical practice.

#### 7.2.1 Clinical investigation of probiotics in combination

Numerous research has examined the effectiveness of probiotics' synergistic effects to ameliorate mood disorders; specifically listed below are the most relevant studies analysed (Table 2). All the studies collected evaluated the change in depressive status through BDI score, while anxiety was measured with BAI score. For example, a study by Akkasheh et al. investigated the impact of probiotic consumption on depressive symptoms. The study assessed metabolic profiles, high-sensitivity C-reactive protein (hs-CRP) levels in serum, and OS biomarkers in individuals diagnosed with MDD.^111^ 40 patients with MDD ranging in age from 20 to 55 years participated in this double-blind, randomized, controlled clinical experiment with a placebo. Patients in the probiotic group (n = 20) were given one probiotic capsule per day that contained *L. acidophilus*, *L. casei*, and *B. bifidum*, which are expressed as colony-forming units (CFU). More specifically, participants were given one probiotic capsule per day that contained *L. acidophilus* (2 × 10^9^ CFU/g), *L. casei* (2 × 10^9^ CFU/g), and *B. bifidum* (2 × 10^9^ CFU/g). To ensure they continued with their regular diet and level of physical activity throughout the intervention, all participants were given three dietary records (two for the weekdays and one for the weekend) and three records of their physical activity. They were also asked not to take any medications that might impact the results during the eight-week intervention. To conduct the analyses and account for the study's secondary outcomes, 10 mL of fasting blood was taken at the study's baseline and endpoint. Patients who got probiotic supplements after an 8-week intervention had a considerably lower overall BDI score than those who received a placebo, which is especially important given that this was the study's main goal. Furthermore, compared to placebo, probiotic supplementation caused a substantial drop in insulin, insulin resistance, and hs-CRP serum levels and a significant increase in plasma glutathione (GSH) levels. However, after supplementation, they did not see appreciable changes in fasting plasma glucose, the quantitative insulin sensitivity check index, lipid profiles, or total antioxidant capacity levels. Probiotic therapy, therefore, had positive impacts on a variety of measures in patients with MDD.^111^ However, it is necessary to consider certain limitations of this study: it was not possible to analyse the effect of probiotic supplementation on other biomarkers of inflammation and oxidative stress; a long-term intervention would be necessary to confirm the beneficial effects of these probiotics on lipid profiles, whereas here they were only administered for 8 weeks; further studies using each of the strains used in this study individually are needed to assess their beneficial effects on symptoms of depression and metabolic status in patients with MDD, and, finally, large-scale studies are needed to confirm the results.^111^ (see Table 3)

**Table 2:** Summary of selected trials analyzing single probiotic strains.

Study type and population	Treatment	Methodology	Results	Ref
44 patients with IBS and mild to moderate anxiety and/or depression scores in a double-blind, placebo-controlled randomized clinical trial	*B. longum*	The patients were subjected to: • HADS evaluation; • State-Trait Anxiety Inventory evaluation; • Birmingham IBS score evaluation; • Bristol stool scale evaluation; • Functional magnetic resonance imaging The blood and the spleen were utilized for: • Serum BDNF analysis; • Serum cytokine analysis; • Serum C-reactive protein analysis.	• Decrease in depression ratings but not anxiety scores. • Improvement in quality of life and overall IBS symptoms. • Changes in the activity of several brain regions involved in processing emotions.	^110^

**Table 3:** Summary of selected trials analyzing the use of probiotic strains in combination.

Study type and population	Treatment	Methodology	Results	Ref
40 patients with MDD, age ranged between 20 and 55 years in a randomized, double-blind, placebo-controlled trial	*L.acidophilus*, *L.casei* and *B. bifidum*	The patients were subjected to: • BDI evaluation; • BMI evaluation. The blood was utilized for: • Fasting plasma glucose analysis; • Lipid concentrations analysis; • Serum C-reactive protein analysis; • Total antioxidant capacity analysis; • GSH levels analysis.	• Significantly lower serum hs-CRP concentrations, insulin levels in the blood, and scores on the BDI's overall scale; • Homeostasis model assessment of insulin resistance. • Values of plasma total GSH significantly increased	^111^
156 healthy adults with subclinical symptoms of depression, anxiety, and insomnia in a randomized, double-blind, placebo-controlled trial	*L.reuteri* NK33 and *B. adolescentis* NK98	The patients were subjected to: • Stress Response Inventory evaluation; • BDI evaluation; • BAI evaluation; • Pittsburgh Sleep Quality Index evaluation; • Insomnia Severity Scale evaluation; The blood was utilized for: • IL-6, TNF-alpha, BDNF, adrenocorticotropic hormone and cortisol analysis.	• IL-6 has been significantly reduced in serum. • Improvement of insomnia symptoms and sleep quality. • Return to a balanced makeup of the gut microbiome.	^112^
110 patients with MDD, age ranged between 18 and 50 years in a double-blind, placebo-controlled randomized clinical trial	*L.helveticus* R0052 and *B. longum* R0175	The patients were subjected to: • BDI evaluation; The serum and urine were utilized for: • TNF-α, IL-1B, IL-6, IL-10 analysis; • Urinary cortisol analysis.	• Enhanced BDI score; • IL-1B levels significantly drop. • Cortisol levels dropped by 20% from baseline. • No change was seen in the levels of serum inflammatory markers.	^113^
71 patients with depressive symptoms in a randomized, triple-blind, placebo-controlled trial	*B. bifidumW*23, *B. lactis* W51, *B. lactis* W52, *L. acidophilus* W37, *L. brevisW*63, *L. casei* W56, *L. salivarius* W24, *L. lactis* W19 and *L. lactis* W58	The patients were subjected to: • BDI evaluation; • BAI evaluation; • Depression Anxiety Stress Scale evaluation; • Cognitive reactive assessment. The stool samples were utilized for: • Microbiome analysis	• A notable decrease in cognitive reactivity of considerable magnitude. • The microbiota was not considerably changed. • Ruminococcus gnavus's correlation with one depression metric	^114^
60 severe depressed patients over 18 years old in a double-blind, controlled randomized clinical trial	*S. thermophilus* NCIMB 30438*, B. breve* NCIMB 30441*, B. lactis* NCIMB 30435*, B. lactis* NCIMB 30436*, L. acidophilus* NCIMB 30442*, L. plantarum* NCIMB 30437*, L. paracasei* NCIMB 30439*, L. helveticus* NCIMB 30440	The patients were subjected to: • BDI evaluation; • HAM-D evaluation; • Gastrointestinal Symptom Rating Scale evaluation; • State-Trait Anxiety Inventory 1 evaluation. The stool samples were utilized for: • Enterotyping analysis; • Diversity measures; • Evaluation of bacterial taxa.	• Significant improvement in depressive symptoms was observed. • A reduction in the HAM-D scores. • Preservation of microbial diversity and augmentation of the prevalence of the genus *Lactobacillus.* • There is a notable reduction in putamen activation when individuals are presented with neutral facial expressions.	^115^
48 patients with GAD in a double-blind, placebo-controlled randomized clinical trial	*B. longum, B. bifidum, B. lactis* and *L. acidophilus bacteria*	The patients were subjected to: • BAI evaluation; • HAM-A evaluation; • WHO- QOL-BREF evaluation; • State-Trait Anxiety Inventory 1 evaluation. The serum samples were utilized for: • Serum cortisol analysis; • ACTH analysis;	• Decrease in the score of HAM-A, BDI and of State-Anxiety Inventory score. • No significant difference in the score of Trait-Anxiety Inventory and quality of life	_116_

The effectiveness of *L. reuteri* NK33 and *B. adolescentis* NK98 in decreasing stress, anxiety, and sleep disturbances, as well as measuring some blood biomarkers, particularly pro-inflammatory cytokines like IL-6, was demonstrated in a double-blind, randomized, placebo-controlled clinical trial that included 156 healthy individuals between the ages of 19 and 65 who had undergone psychological stress and had subclinical symptoms of depression or anxiety. Furthermore, it was presumed that they had not received any stress, depression, or anxiety therapy in the preceding four weeks. Two capsules of water were to be taken once a day by the patients in the intervention group (n = 78). The NVP-1704 capsules were composed of microorganisms, each 500 mg capsule containing 2.5 × 10^9^ CFU. Specifically, *L. reuteri* NK33 contributed 2.0 × 10^9^ CFU, while *B. adolescentis* NK98 contributed 0.5 × 10^9^ CFU. The key results of this study were the BDI-II and BAI scores, both of which decreased after four weeks of treatment. Although no differences were observed in the change of serum BDNF levels and no significant inter- or intra-group differences were observed following interventions in other blood biomarkers like TNF-α, ACTH, and cortisol, some differences in serum IL-6 concentrations between the two groups were identified in detail. Furthermore, administration of NVP-1704 tended to increase weakly both α-diversity (estimated operational taxonomic unit richness), which refers to the abundance of strain types, and β-diversity, which refers to species diversity in the composition of the microbiota: NVP-1704 administration increased the *Actinobacteria* population and tended to decrease the *Proteobacteria* population while significantly increasing the *Bifidobacteriaceae* population. The Pittsburgh Sleep Quality Index and the Insomnia Severity Scales, used in this study to measure the severity of insomnia symptoms, show a substantial decrease in symptoms associated with probiotic medicine for the first time.^112^ Nonetheless, future research involving large-scale, closely monitored, long-term human trials should be considered to verify the positive benefits of different probiotics on mental well-being and sleep.

In contrast, a double-blind, placebo-controlled experiment by Kazemi et al.^113^ examined the impact of an 8-week probiotic supplement on pro-inflammatory cytokines in a sample of 110 MDD patients aged 18 to 50. Three experimental methods were used to divide the participants: prebiotic, probiotic, and placebo. The prebiotic product comprised galactooligosaccharide with 0.2% plum flavour, whilst the probiotic group received a sachet containing 10 × 10^9^ CFU of freeze-dried *L. helveticus* R0052 and *B. longum* R0175. This study specifically demonstrated how supplementation with *L. helveticus* R0052 and *B. longum* R0175 reduced depression while not affecting inflammation according to the evaluated non-significant changes in levels of all pro-inflammatory markers. Specifically, the probiotic-treated group experienced a significant decline in BDI scores, while all groups had comparable cytokine levels. However, the patients were not taking the same antidepressant, so the study results may not be homogeneous. In addition, the recruitment phase lasted a long time. It was conducted at different times of the year, and although the conditions were identical for all three experimental groups, changes in lifestyle, diet, vitamin D status, and seasons could dilute the effect of the probiotic.

Another randomized, triple-blind, placebo-controlled trial^114^ that included 71 people and a comparable probiotic mixture also examined the effects of the two probiotic combinations when taken daily for eight weeks. For the probiotic therapy, a combination of *B. bifidum* W23, *B. lactis* W51, *B. lactis* W52, *L. casei* W56, *L. salivarius* W24, *L. lactis* W19, *L. acidophilus* W37, *L. brevis* W63, and *L. lactis* W58 was administered to see how their consumption affected a sample of participants with mild to severe depression. Only the probiotic group's individuals showed a significantly lower level of cognitive reactivity, even though all participants showed improvement in depressive symptoms (as judged by the BDI and BAI scores). Furthermore, no variations in alpha or beta diversity within the microbiota between pre-and post-treatment samples, within the placebo and probiotic groups, or between groups were discovered. Testing for variations in the relative abundance of bacterial taxa in this situation revealed no discernible differences between groups at the pre- or post-treatment time points. Nevertheless, even though the microbiota of depressed people did not significantly change, a strong association was discovered between *Ruminococcus gnavus* and one depression metric. However, some research contends that a new strategy for treating or preventing depression might entail changing the microbial ecology of the human stomach by introducing probiotics. Future studies should look at different analytical techniques to investigate specific strains of the gut microbiota and how to use probiotics most effectively, for instance, by adjusting doses and timing. Furthermore, Schaub et al. examined neuronal alteration and microbiome change in 60 patients experiencing depressive episodes.^115^ This phenomenon was observed after a regimen of probiotic administration characterised by a brief duration and high dosage. The participants assigned to the probiotic group were administered a supplement comprising eight different strains, namely *S. thermophilus* NCIMB 30438, *B. breve* NCIMB 30441, *B. longum* NCIMB 30435 (recently reclassified as *B. lactis*), *B. infantis* NCIMB 30436 (recently reclassified as *B. lactis*), *L. acidophilus* NCIMB 30442, *L. plantarum* NCIMB 30437, *L. paracasei* NCIMB 30439, and *L. delbrueckii subsp. Bulgaricus* NCIMB 30440 (recently reclassified as *L. helveticus*). The daily dosage of the supplement contained a total of 900 billion colony-forming units daily. After the therapy, the Hamilton Rating Scale for Depression (HAM-D) scores in the probiotics group showed a faster decline over time, with an increased abundance of the *Lactobacillus* and a reduction in depressed symptoms. These findings demonstrated that probiotic supplementation may help reduce depressed symptoms, alter the microbiome, and highlight the gut-brain axis. Although no statistically significant alterations were observed in alpha-diversity measures over time within both the probiotics and placebo groups, comparing the two groups at post-intervention and follow-up revealed that probiotics exhibited sustained diversity. In contrast, the placebo group experienced a decrease in a distinct index but not in observed richness. Between study groups, there were noticeable disparities in beta diversity, according to the results; however, the sample size is relatively small, and compliance was not perfect; cases with poor compliance were excluded. Thus, large-scale research is required to confirm and reproduce the findings. Additionally, it would be crucial to examine the interactions of probiotics with general antidepressant drugs to determine whether there are generalized benefits or probiotics' positive effects depending on a particular antidepressant. Considering the briefness of the four-week timeframe, it would also be intriguing to see if modifications in brain structure and function become more noticeable following the intervention.

The HAM-D score, as well as the State-Anxiety Inventory score, decreased after the consumption of *B. longum*, *B. bifidum*, *B. lactis*, and *L. acidophilus* (18 × 10^9^ CFU) for 4 weeks of intervention with sertraline (25 mg) for 8 weeks. Still, this intervention did not result in statistically different results from the placebo group. This study^116^ further examined probiotics' effects on anxiety-related symptoms. A statistically insignificant difference between the two groups was not seen, nor did either group's plasma ACTH or serum cortisol levels alter appreciably. Despite showing that sertraline plus probiotics reduced anxiety symptoms more effectively than sertraline alone after eight weeks, this medication had no adverse effects on quality of life. It is, however, suggested that future clinical studies should investigate the intestinal colonisation of probiotics in favour of more complete data.

In addition to these findings, Chao et al.'s meta-analysis showed that the impact of probiotics on the human body varies from person to person and parameter to parameter, with different responses for anxiety and depressive states. Probiotics may be used as adjuvant therapy for emotional or mood disorders because they alleviate depressive symptoms in participants both with and without a clinical diagnosis or in the absence of other therapeutic options. Thus, microbiota-based interventions with probiotics may possess greater therapeutic potential for depression treatment, which can be used as an adjunct to current approaches. Therefore, probiotics should be utilized more frequently in treating depression.^117^ Ng et al. meta-analysis found that patients with mild to moderate depression benefited statistically significantly from probiotics, in contrast to Chao et al. meta-analysis's finding that the overall impact of probiotics on depressed and healthy adults was statistically negligible.^118^ These findings contrast significantly with a meta-analysis by Huang et al. who compiled information from 5 randomized controlled trials and discovered that probiotics' benefits on mood were statistically significant in healthy and depressed people.^119^ Due to differences in probiotic doses and therapy durations between studies, clinical trial consistency has also been affected, hindering the ability to draw meaningful conclusions from meta-analyses. Similarly, variable use of bacterial strains and strain combinations is expected to impact trial outcomes because some bacteria have been shown to have stronger anti-depressant effects than others.^120^ Probiotic supplementation appears to have had a good effect on lowering anxiety and depression symptoms in several trials despite the current research's limitations and/or inconsistent outcomes. Nevertheless, because most of the available clinical trials were conducted on healthy individuals, extrapolating the present findings to those with depressive disorders is challenging. These results should be considered preliminary until further research considers the complexity of the human gut microbiota, gut-brain axis, and CNS interactions, particularly concerning particular probiotic strains.

#### 7.2.2 Clinical investigation of probiotics in combination with natural compound

In addition to the study of different combinations of probiotic strains, there are several studies in the literature about the effect of combinations of probiotic strains with natural substances (Table 4). For instance, the study conducted by Reininghaus et al.^103^ proves the significance of vitamins in modifying symptoms related to mood disorders. In this study, which followed a monocentric, randomized, placebo-controlled design, the probiotic group (n = 42) received a combination of *B. bifidum* W23, *B. lactis* W51, *B. lactis* W52, *L. acidophilus* W22, *L. casei* W56, *L. paracasei* W20, *L. plantarum* W62, *L. salivarius* W24, and *L. lactis* W19 along with Vitamin B7 (125 mg) for 28 days. On the other hand, both groups, including the placebo group (n = 40), were administered Vitamin B7 (125 mg) for the same 28-day period. Both cohorts made significant improvements regarding psychiatric symptoms, and there was no discernible difference between the probiotics and placebo groups regarding changes on any of the psychiatric scales. After the study, they also discovered a significant rise in the taxonomically related *Coprococcus* 3 in the probiotics group. This information is crucial because, according to recent research, *Coprococcus* bacteria is consistently linked to better quality of life indicators. *Coprococcus* species are depleted in people who suffer from depression.^121^ Moreover, the intervention group showed upregulation in vitamins B6/B7 and B1 metabolism. This finding holds significant importance as it is well-established that vitamin B6 metabolism is implicated in the pathophysiology of psychiatric disorders. Additionally, the active form of vitamin B6, pyridoxal 5′-phosphate (PLP), is crucial in regulating plasma homocysteine levels.^122^ It is also likely that some of the bacteria in the probiotic product increased the availability of biotin in the gut since both study participants received the same amount of vitamin B7 (biotin). Still, only those receiving a probiotic supplement appear to have their metabolism under control. The verum and control groups also differed considerably regarding several metabolic pathways, including glucose and fat metabolism. This study is significant since many people with depression have somatic comorbidities, which lower quality of life and drastically shorten life expectancy. One of the most important findings of the study was an increase in the IL-17 pathway, which was shown to be the most potent effect induced by the probiotic intervention because IL-17 is a critical mediator of inflammation and plays a key role in immune activation and, consequently, in autoimmune diseases.^123^ This study provides evidence that probiotic administration may help patients with depressive disorders balance their microbiome composition in addition to conventional therapy.^103^ Nevertheless, this study has some limitations, such as the relatively small sample size, the intake period, which may have been too short to observe changes at the clinical level, the hospitalisation of the patients, which may have changed their eating habits, and the difference in smoking status at baseline between the two groups, which may have had a confounding influence on the results. Finally, due to the large number of women in the study, the results may reflect the situation in women more closely than in men.

**Table 4:** Summary of selected trials analyzing probiotic strains in combination with natural compound.

Study type and population	Treatment	Methodology	Results	Ref
82 patients with depression in a randomized, double-blind, placebo-controlled trial	*B. bifidum* W23, *B. lactis* W51, B*. lactis* W52, *L. acidophilus* W22, *L. casei* W56, *L. paracasei* W20, *L. plantarum* W62, *L. salivarius* W24 and *L. lactis* W19 along with Vitamin B7	The patients were subjected to: • BDI evaluation; • HAM-D evaluation; • Symptom Checklist-90-Revised evaluation; • Mania Self Rating Scale evaluation; • Gastrointestinal quality of life questionnaire The stoll samples were utilized for: • Microbiome analysis (16 S rRNA-sequencing)	• Heightened activation of inflammation-regulatory and metabolic pathways. • Production of vitamin B6 and B7 is increased. • IL-17 pathways are upregulated.	^103^
75 patients in hemodialysis with depression and anxiety symptoms in a randomized, double-blinded, clinical trial	*L.acidophilus T16* *B. bifidum BIA-6,* *B. lactis BIA-7, and B. longum BIA-8 plus fructo-oligosaccharides, galacto-oligosaccharides and inulin in the synbiotic group.*	The patients were subjected to: • HADS; • Gastrointestinal Symptom Rating Scale assessment. The serum and stoll samples were utilized for: • Serum BDNF analysis; • Fecal colony counting.	• Improvement in serum BDNF levels and depression symptoms in the synbiotic group.	_124_

Following supplementation with *L. acidophilus* T16, *B. bifidum* BIA-6, *B. lactis* BIA-7, and *B. longum* BI-8, the symptoms of depression were also evaluated in hemodialysis patients.^124^ In this study, a randomized, double-blinded clinical trial involving 75 patients was conducted. These patients were assigned to different treatment groups, namely the synbiotic, probiotics, or placebo groups. The synbiotic group received a combination of 15 g of prebiotics and 5 g of probiotics, which contained specific strains, including *L. acidophilus* T16, *B. bifidum* BIA-6, *B. lactis* BIA-7, and *B. longum* BIA-8 (each with a concentration of 2.7 × 10^7^ CFU/g). The probiotics group received the same 5 g of probiotics as the synbiotic group but with an additional 15 g of maltodextrin as a placebo. Lastly, the placebo group received 20 g of maltodextrin. All treatments were administered for 12 weeks. Even though probiotic supplementation did not significantly reduce depressive symptoms, a substantial impact was observed following symbiotic supplementation with prebiotics. All patients showed signs of this symbiotic relationship with anxiety symptoms. Furthermore, the symbiotic group's serum BDNF level considerably increased compared to the other groups, indicating a neuroinflammation-related effect. In addition, using the synbiotic or probiotic supplements affected the amount of faecal *bifidobacteria* and lactobacilli colonies while lowering the number of coliform colonies during the 12-week trial period. This contrasted with the placebo group. Further studies evaluating the impact of prebiotics, probiotics, or synbiotics on blood BDNF levels in hemodialysis patients may shed more light on the processes behind this probiotic combination's antidepressant and antianxiety effects.

## 8 Effectiveness of probiotics on human health

Adverse effects are not sufficiently evaluated or consistently recorded, and interventions are poorly documented in probiotic intervention trials.^125^ Even if there have been a lot of publications, the current body of knowledge is insufficient to address concerns regarding the safety of probiotic therapy.^126^ Additionally, unusual adverse events are difficult to analyse. However, in 2014, an international consensus declaration recognised the normalisation of pathological gut microbiota, management of intestinal transit, competitive exclusion of pathogens, and production of SCFA.^94^ However, the consensus panel also noted that many of the additional effects linked to particular probiotics are species- and strain-specific in several medical conditions. In contrast to the likelihood that neurological, immunologic, and metabolic effects may vary depending on dose and strain, vitamin synthesis and gut barrier reinforcement are likely species-specific mechanisms.^94^ Furthermore, it might not be as simple as it initially seems to define a typical microbiota. While there may not be a single healthy condition, it is generally agreed that a “healthy” microbiota has a high level of microbial diversity, a balanced ratio of *Bacteroidetes* to *Firmicutes*, a high level of fecal butyrate, and a low abundance of *C. albicans*.^127^

In this regard, a systematic review by Ng et al.^125^ notes that mental illnesses are a complex and diverse group of conditions and that it typically takes two to three months of taking a common antidepressant drug for symptoms to start to improve and probably longer to achieve a state of clinical remission. Similarly, it may require the host's microbiota to exhibit significant molecular changes for more than eight weeks (the normal research timescale. A brief probiotic therapy may result in a transient and temporary change in the gut flora. In contrast, recent randomized controlled research by Schaub et al.^115^ examined whether short-term, high-dose probiotic therapy decreased depressive symptoms and microbial alterations in the gut and brain in depressed individuals. As already established, trial subjects were randomly assigned to take a placebo for 31 days or a probiotic multi-supplement. The treatment for depression episodes was also ongoing for the patients. The outcomes illustrated the promise of microbiota-related therapeutic approaches as beneficial, affordable, and non-stigmatizing treatments for mood disorders by illuminating the efficacy of supplemental probiotic therapy in lowering symptoms of depression. There were also modifications in the microbiomes of the brain and intestines. They hypothesized that probiotics maintained microbial diversity and increased the abundance of the chosen genus (in this case, *Lactobacillus*), demonstrating the effectiveness of probiotics in boosting certain taxa. Increases in *Lactobacillus* were specifically linked, in the probiotic group, to a reduction in depressive symptoms. This aligns with the notion that the gut microbiota's makeup has noticeably changed eight weeks after the treatment. This shows that taking a daily dosage of probiotics could be an effective alternative for managing the signs of anxiety and depression. However, it could not determine probiotics' fleeting and ephemeral nature because no studies could be found examining their effects and persistence after therapy was completed.

Overall, many preclinical and clinical investigations have been conducted on the intestinal advantages of probiotics in healthy individuals and a wide range of minor and serious health disorders. Furthermore, it is important to acknowledge that a significant proportion of commercially available probiotics are sourced from fermented foods that have a well-established record of safe consumption or from microorganisms that have the potential to inhabit the bodies of healthy individuals.^128^^,^^129^ Additionally, it is worth mentioning that most probiotic clinical trials documented in the existing literature have not identified any significant safety issues.^118^ The absence of systematic records of adverse events and the lack of trustworthy long-term data prevents solid long-term predictions^127^ even though the available studies do not indicate an increased risk of serious side effects. According to anecdotal reports, probiotics may have negative effects: faecal microbiota transplant patients frequently complain of gastrointestinal issues such as diarrhea, bloating, and abdominal pain 130. These reports will significantly impact the management of both the short- and long-term.^131^^,^^132^ Therefore, further research is needed to fully understand probiotics' efficacy in treating depression, even though the extensive evidence from currently available clinical trials does not show an increased risk in their use.

A summary of probiotic's strengths and weaknesses in modulating anxiety and depression is reported in Table 5.

**Table 5:** Summary of advantages and disadvantages related to probiotics use.

**Advantages**	Microbiota-related therapeutic approaches can be beneficial, accessible, and non-stigmatizing treatments for mood disorders.^115^
	Probiotics maintain microbial diversity and increase the abundance of the chosen genus.^115^^,^^127^
	Increases in *Lactobacillus* were specifically linked to a reduction in depressive symptoms.^115^
	Available studies do not indicate an increased risk of serious side effects.^118^
**Disadvantages**	Probiotics likely have species-specific mechanisms that may vary depending on dose and strain ^94^.
	A brief probiotic therapy may result in a transient and temporary change in the gut flora.^125^
	No studies have been conducted on the effects and persistence of probiotics after the end of therapy.
	Some anecdotes have recorded negative effects about probiotics use: patients undergoing fecal microbiota transplantation often complain of gastrointestinal problems such as diarrhea, bloating, and abdominal pain 130,131.

It must also be considered that professional and commercial food supplements containing probiotics are widely available. It is also challenging to determine whether the product matches the one used in the research since, regrettably, research on the designation of medicinal strains is inconsistent, and sometimes, the strains need to be mentioned on the labels of the supplements. This is also because strain identification requires a combination of phenotypic and genotypic testing. Still, species identification is unregulated, and supplement manufacturers are not obligated to provide this information on labels.^132^ In this context, a study by Dolan et al. analysed the composition of several commercial products used in the framework of mental health, including anxiety and depression. This study showed several commercial products on the market containing different probiotic strains. Among these for anxiety were several products containing the probiotic strains analysed in the study by Messaoudi et al. described above.^101^ Specifically on the market, products containing *L. helveticus* and *B. longum* include Xymogen Probio Defence and Pure Encapsulations. In contrast, studies by Akkasheh et al.,^111^ showed that the combination of *B. longum*, *B. bifidum*, *B. breve*, *B. infantis*, *L. helveticus*, *L. rhamnosus*, *L. acidophilus*, *L. casei* and *L. plantarum* strain PS128 has been used to make several commercial products such as Primadophilus Optima and RAW Probiotics Ultimate Care. From these studies^111^ and the use of this combination, several products for managing depression have also been developed; among these can be named Probiophage DF, Xymogen Probio Defense and FlorAssist Probiotic.^132^

## 9 Postbiotics are a booster for mental health

**Fig. 5:**
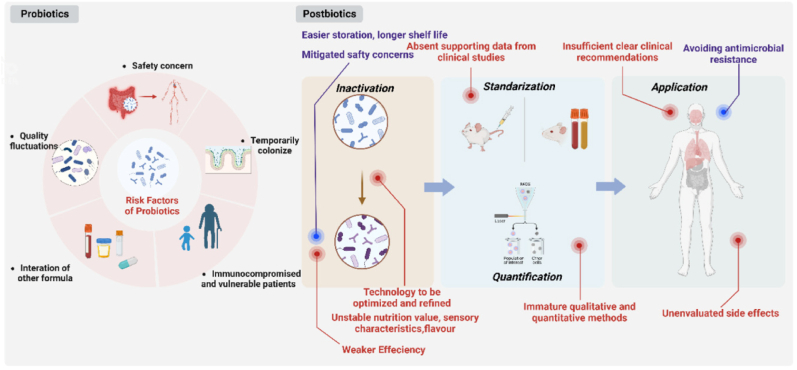
Comparison of probiotics and postbiotics application^133^.

Recently, postbiotics, a new member of the biotic family that directly or indirectly enhances the host's health, have come to light.^133^ The term “postbiotic” was officially defined by the International Scientific Association for Probiotics and Prebiotics (ISAPP) in the year 2021 to indicate “a preparation of inanimate microorganisms and their components that confers a health benefit on the host”^134^ combining the Greek words ‘post’ (after) and 'bios' (life).^135^ Postbiotics are chemical substances with a bigger molecular weight (such as bacteriocins) than short- and long-chain antibacterial molecules (such as carbon dioxide, diacetylene, and hydrogen peroxide). Additionally, postbiotics are soluble substances secreted or released by bacteria after lysis (such as enzymes, peptides, teichoic acids, muropeptides made from peptidoglycans, polysaccharides, cell surface proteins, etc.). These immunity boosters aid in the reduction of inflammation brought on by pathogens and support intestinal epithelial cells' capacity to survive.^136^ Postbiotics are significantly safer than probiotics in terms of protection (Fig. 5). Indeed, unlike probiotics, they avoid issues related to absorbing virulence factors and genes for antibiotic resistance.^137^

Since children have an underdeveloped and feeble immune system and a porous intestinal barrier, various studies have shown that they are protected against live germs throughout their early development. Postbiotics are an effective replacement for therapies for several allergy illnesses because they can enhance immune system maturation by re-establishing the equilibrium between T helper cells 1/T helper cells 2 (Th1/Th2) mediated immunity.^138^ Additionally, postbiotics are frequently considered to possess positive traits like a clear chemical structure, a longer shelf life (up to 5 years), and safe dose limits.^139^ Even though it is established that postbiotics have positively affected health, the exact mechanisms are not yet understood. Due to substances that imitate probiotics' advantageous and therapeutic effects, postbiotics may have a protective effect even if their modes of action differ. Indeed, by reducing metalloproteinase-9 activity, postbiotics generated from *Lactobacillus* have been shown to limit colon cancer invasion. Furthermore, *B. infantis*, *B. longum*, *B. adolescentis* and *B. breve* all produce nicotinamide adenine dinucleotide oxide (NADH) peroxidase, which has the potential to break down hydrogen peroxide.^140^ Concerning mental illnesses, anxiety, and despair, this innovative treatment approach has found a significant demand. Since 2009, there has been consistent reporting in the literature of scientific evidence indicating that inactivated microbes have a beneficial effect on human health.^141^

The development of postbiotics is largely fuelled by fermentation, according to numerous studies. Microbial cells utilise prebiotics during fermentation to create some postbiotics from their source components. They naturally produce a range of postbiotic chemicals with different biological qualities (antimicrobial, antioxidant, anticancer, etc.), which enriches the food matrix with these advantageous molecules.^142^

Due to their advantages for biotherapeutic application, postbiotics have a safe profile, well-known chemical structures, hydrolysis resistance, nontoxicity, digestive system resilience, and prolonged shelf life. Huge efforts are being undertaken to anticipate using postbiotics as biotherapeutic agents for eradicating various diseases.^138^ The psychobiotic effects of probiotic diets have been evaluated in several clinical studies about this. Over 12 weeks, Chung et al. studied the psychobiotic effects of fermented milk pills containing *L. helveticus* IDCC3801 in healthy elderly individuals aged 60 to 75. Fermented milk tablets improved patients' cognitive function compared to the placebo group. Similar to that, for eight weeks, 61 healthy middle-aged individuals (50–70 years old) received fermented milk containing *L. helveticus* CM4 and lactononadecapeptide.^143^^,^^144^

Considering the state of contemporary medicine, the coronavirus disease 19 (COVID-19) pandemic claimed the lives of over three million individuals in less than a year. Aside from the death toll, 150 million survivors experienced emotional, physical, and financial challenges, which resulted in vicarious traumatization, which causes hopelessness, anxiety, and post-traumatic stress disorder (PTSD).^145^

Probiotics may alleviate PTSD brought on by COVID-19; in particular, the *Bifidobacterium* is the most widely used probiotic in preventing and treating depression. Bifidobacteria can be used to treat depressive symptoms, may be related to reducing the abundance of pathogenic bacteria, exerting anti-inflammatory effects, improving the permeability of the intestinal barrier, regulating the tryptophan levels, affecting 5-hydroxytryptamine (5-HT) synthesis, and regulating hypothalamus–pituitary–adrenal (HPA) axis.^146^

On the other hand, *L. helvetius* may worsen cognitive deficiencies, including memory loss and loss of focus, by releasing serotonin and NA through controlling the central serotonin system, NA system, and HPA axis. Additionally, the histamines produced by *L. reuteri* in intestinal epithelial cells reduce the expression of pro-inflammatory cytokines, which halts the depletion of BDNF, a biomarker for mental health in the hippocampal region.^146^

**Fig. 6:**
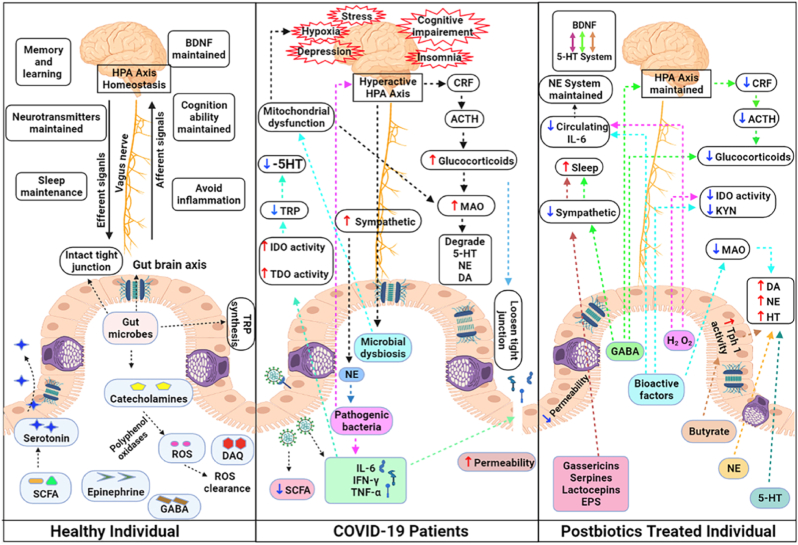
Postbiotics in balancing hypothalamic–pituitary–adrenal axis and mental health during post-COVID regime.^134^**BDNF**: brain-derived neurotrophic factor; **DA**: dopamine; **5-HT**: 5-hydroxytryptamine or serotonin; **BZA**: benzoic acids; **dgk**: diacylglycerol kinase; **EPS**: exopolysaccharide; **GABA**: gamma-Aminobutyric acid; **GLP-1**: glucagon-like peptide,1; **Glu**: glutamate or glutaminergic; **H**_**2**_**O**_**2**_: hydrogen peroxide; **HPA**: hypothalamic–pituitary–adrenal axis; **IECs**: intestinal epithelial cells; **IDO**, indoleamine 2,3-dioxygenase; **IL-6**: interleukin-6; **KYN**: kynurenine; **NE**: norepinephrine; **ROS**: reactive oxygen species; **SCFA**: short-chain fatty acid; **Tph1**: tryptophan hydroxylase 1; **TRP**: tryptophan.

SCFA, like butyrate, are also necessary for maintaining the integrity of the gut barrier, which affects the expression of BDNF and the modulation of the serotonin pathway in the CNS. Stress and anxiety are protected from the body by a decrease in inflammation brought on by managing SCFA in the brain. Various postbiotic compounds produced by specific probiotic strains play a key element in neuromodulation activities (Fig. 6, 134) produced by *B. longum,* which influences brain inflammation by reducing proinflammatory signals. In addition to improving the composition of the gut microbiome and inducing sleep, *L. gasseri* also produces gassericins. During viral infection, lactocepins produced by *L. paracasei* help reduce inflammatory cytokine migration by maintaining intestinal epithelium integrity.^146^ *B. infantis* produces similar polysaccharides, increasing the CNS's production of NE neurotransmitters, while *L. kefiranofaciens* secretes extracellular polysaccharides that have immunomodulatory qualities that may reduce the HPA axis's hyperactivity^146^

Additionally, several probiotic strains generate postbiotic compounds that are key in neuromodulation processes. In a recent clinical experiment, 60 adult students were randomly divided into two groups and given postbiotics twice daily or a placebo for 24 weeks. The results included changes in the quantities of SCFA in the faeces, salivary cortisol, and faecal microbiota studies. SCFA and faecal microbiota levels were analysed using Analysis of Covariance, demonstrating significant differences (p < 0.05) between the two groups.^147^ Additionally, in young adults with persistent psychological stress, *L. gasseri* CP2305, has shown efficacy and long-term health advantages. Indeed, when compared to a placebo, *L. gasseri* CP2305 considerably reduced anxiety and sleep disturbance, according to studies employing questionnaires to assess mental and physical states. Additionally, faecal microbiota research shows that adding *L. gasseri* CP2305 to the diet prevented stress from increasing *Streptococcus* spp. and decreasing *Bifidobacterium* spp*.*^147^

Another cohort study reported that the higher relative abundances of *Faecalibacterium* and *Coprococcus* were associated with a higher quality of life, and lower abundances of *Coprococcus* and *Dialister* were linked to depression. In this context, a correct probiotic supplementation may be useful to reduce the symptoms. Closely related to the concept of postbiotics are bioactive metabolites, sometimes referred to as biogenics, which are produced by bacteria during fermentation processes. These fermentation products include, for example, vitamins (such as B vitamins), bioactive peptides (such as lactotripeptides), bacteriocins (which aid with bacterial survival), short-chain fatty acids (such as butyric acid), and neurotransmitters (such as GABA [gamma-aminobutyric acid] and serotonin).^148^ Postbiotic supplementation does not reveal side effects during human administration.^147^

**Fig. 7:**
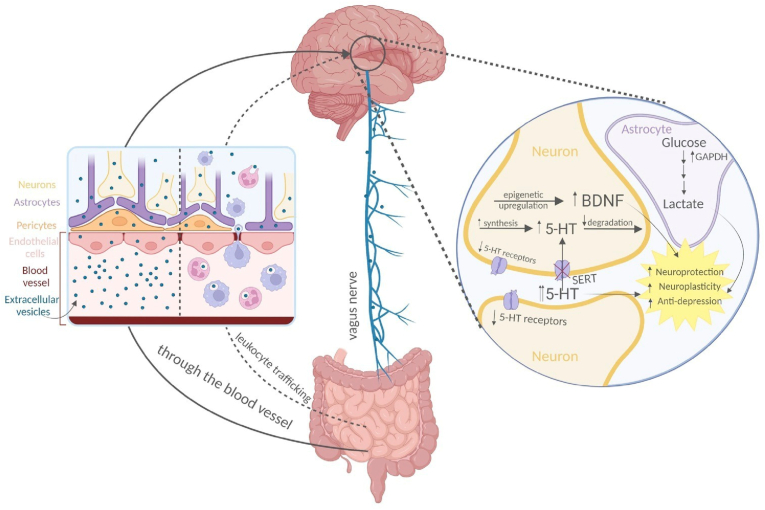
Schematic representation of the effects of postbiotics linked to brain tissue. The figure reported is taken by Bleibel et al..^149^**BDNF**: brain-derived neurotrophic factor; **5-HT**: 5-hydroxytryptamine or serotonin; **GAPDH**: glyceraldehyde-3-phosphate dehydrogenase.

The host benefits from postbiotics, although the precise processes by which this occurs are not still understood. Furthermore, postbiotics have been studied in the food industry in addition to *in vitro* and *in vivo* research, with evidence indicating that they have a longer shelf life than another biotics and are simpler to store and handle. Postbiotics might be a useful therapeutic approach. This research will benefit human patients and open the way to creating novel pharmaceutical and dietary products with specific physiological effects, summarized in Fig. 7.

## 10 Conclusions

In the area of mental health, probiotics have proven advantages and promise. Supplemental probiotics, in particular, alter the gut-brain axis, creating neurotransmitters, an anti-inflammatory response, and epigenetic mechanisms. The regulation of the gut microbiota by probiotic supplementation may be advantageous since the multiple metabolic functions these supplements stimulate also coincide with the recovery of the pathophysiological pathways for depression. However, the therapeutic benefits of probiotics on mental health have not yet been thoroughly examined despite a wealth of preclinical data. As a result, more research is required to establish the effectiveness of probiotics in reducing depressed symptoms and the appropriate treatment time, dosage, and strain of probiotics to achieve efficacy in mental health.

Regulators need an upper limit on the number of live microorganisms permitted to remain following postbiotic processing. According to the inactivation conditions, most postbiotic products will include some survivors. Different inactivation methods and technologies, such as heat, high pressure, and oxygen exposure duration for anaerobic microbes, might leave behind variable numbers of live cells of the progenitor microorganisms. When considering the trade-off between safety considerations and performance disparities in probiotics and postbiotics, it becomes imperative to promptly suggest a compromising and optimal prescription tailored to the patient's unique condition. This may involve judiciously utilising both probiotics and postbiotics in suitable proportions.

Nevertheless, our study has certain limitations because of the high heterogeneity of the probiotics included. It must, however, be considered that the use of probiotics to date has no defined guide, and there is no elective choice for a specific disease. Hence, the heterogeneity of probiotics under consideration proves to be a strength.

## Author contributions

Investigation, F.S. and M.S.; data organization, G.R, P·F., B.A. R.S. and R.G.; writing—original draft preparation, F·S., M.S., G.R, P·F., R.S and M.C.; writing—review and editing, R.S., M.C. and U·F; supervision, U.F. All authors have read and agreed to the published version of the manuscript.

## Funding

This research received no specific grant from funding agencies in the public, commercial, or not-for-profit sectors.

## Declaration of competing interest

All the authors declare no conflict of interest.
